# Three-dimensional quantitative assessment of palatal bone height for insertion of orthodontic implants - a retrospective CBCT study

**DOI:** 10.1186/s13005-019-0193-9

**Published:** 2019-04-01

**Authors:** Sachin Chhatwani, Viola Rose-Zierau, Bassel Haddad, Mohammed Almuzian, Christian Kirschneck, Gholamreza Danesh

**Affiliations:** 1 Department of Orthodontics, University of Witten/Herdecke, Dental Clinic, Alfred-Herrhausen Str. 45, 58455 Witten, Germany; 2 Viola Rose-Zierau, Private Practice, Nelkenstr. 2, 44289, Dortmund, Germany; 3 Glasgow Dental Academy, Edinburgh, UK, Department of Orthodontics, University of Sydney, Sydney, NSW Australia; 4 Department of Orthodontics, University Medical Centre of Regensburg, Franz-Josef-Strauss-Allee 11, 93053 Regensburg, Germany

**Keywords:** CBCT, Palatal insertion, Orthodontic implant, Bone height, Skeletal anchorage

## Abstract

**Background:**

Orthodontic implants have found widespread use as means of maximum skeletal anchorage in fixed orthodontic treatment, their optimal insertion location in the hard palate, however, is still controversial. The aim of this study was therefore to assess mean bone height across the hard palate and possible age- and sex related differences to identify the most favourable location according to maximum bone height, optimizing primary stability and survival of inserted orthodontic implants.

**Methods:**

In this retrospective cross-sectional study, maxillary pretreatment CBCT scans of 180 healthy orthodontic patients (95♀, 85♂, age 8–40 years) were analysed with regard to vertical palatal bone height in the midpalatal area at 88 validated points distanced 2 mm from each other forming a grid of 0–14 mm posterior to the incisive foramen and 10 mm lateral of the midpalatal suture. Differences in bone height regarding sex and topographical location were assessed by three-way ANOVA.

**Results:**

In general, the midpalatal suture as well as the anterior-lateral palatal region showed distinctly higher mean palatal bone height with its maximum 4 mm posterior of the incisive foramen, whereas bone height was limited at the posterior region of the midpalatal suture. Women generally had significantly decreased palatal bone height compared to men at all measurement points. Higher age was associated with a decrease of bone height in the anterior and posterior lateral palatal region and the median palatal raphe with significant age differences.

**Conclusions:**

The midpalatal suture as well as the anterior lateral palate seem to be most suitable for the insertion of orthodontic implants. Palatal bone height, however, was found to be sex- and age-specific, thus sex- and age-related differences should be taken into account, particularly regarding implant length. The ideal insertion site in the palate with sufficient bone height for orthodontic implants is 0-8 mm (men) or 0-6 mm (women) posterior to the incisive foramen and 10 mm lateral to the midpalatal suture.

**Trial registraion:**

This study has been registered and approved by the Ethics Committee of the University of Witten/Herdecke, Germany (12/2016).

**Electronic supplementary material:**

The online version of this article (10.1186/s13005-019-0193-9) contains supplementary material, which is available to authorized users.

## Introduction

The development and clinical usage of orthodontic implants (OI) as means of (maximum) skeletal orthodontic anchorage has had a major impact on orthodontic therapy and extended the scope of treatment possibilities [26]. OI are usually inserted into the buccal and palatal interradicular spaces of the maxilla and mandible, various palatal regions, the retromolar area in the lower jaw as well as the subnasal, symphysis and maxillary tuberosity region [26]. Each region has its potential anatomical and functional advantages and limitations, resulting in increased or limited implant stability and survival.

In general, the maxilla has been shown as particularly favourable for OI insertion. Although various recommendations for the optimal implant location in the maxilla exist [33], the palate is generally considered to be ideal for OI insertion being far from the roots of the teeth, rich with attached gingiva, functionally stable without ample deformation or muscular strain and allowing easy access and topography for OI insertion [19] with orthodontic success rates exceeding 90% [30]. Additionally CAD/CAM guided implant insertion could be advantageous in terms of control and safety during implantation [6], especially in cases with palatally displaced canines.

Regarding the ideal palatal insertion site of OI there is much disagreement in current scientific literature. Some authors report the midpalatal suture of the palate to be an ideal location [2, 14, 24], whereas others consider the paramedian region to be the preferrable insertion site [4, 15, 20, 21, 33]. Currently, there is also disagreement regarding the exact positioning in the paramedian region [15]. OI insertion location, however, is a major determinant for risk of implant loss [3, 17, 27] and thus orthodontic success. Deguchi et al. reported a success rate of 97% in dogs [8], which was higher than reported by a recent systematic review with an overall failure rate of OI of 13.5% (95% CI 11.5–15.9) [1]. OI failure is mainly due to limited primary and secondary stability [26]. Primary implant stability, the pivotal factor in OI success [23], depends not only on bone quality, but also the thickness of cortical bone of the particular insertion site [32]. Sufficient cortical bone thickness allows the OI to be firmly anchored within the bone and to withstand orthodontic loads.

Hence, for palatal insertion, implant sites with a maximum of palatal bone height are favourable regarding primary implant stability and should thus minimize the risk of implant failure maximizing orthodontic success.

In a previous study, Hourfar et al. [16] could show sufficient palatal bone height for implant insertion in the area of the third palatal rugae using cone beam computed tomography (CBCT) scans of the maxilla. Their study, however, did not differentiate patients according to age or sex. A recent search of literature revealed a study correlating sex and growth variation regarding palatal bone thickness based on only eight measurement points in the palate [34]. Holm et al. also differentiated between male and female patients and based their measurements on 40 measurement points projected on the palate [15].

The aim of this study was therefore to quantify palatal bone height with 88 measurement points in the midpalatal region and to assess possible differences by patient age or sex to contribute to evidence-based clinical decision-making, when choosing the optimal OI insertion site. The null hypothesis of this study is that there is no difference in terms of palatal bone height of the midpalatal region between patients of different age and sex groups.

## Material and methods

### Study design, setting and participants

In this retrospective cross-sectional CBCT-scan-based study, which was reviewed and approved by the Ethics Committee of the University of Witten/Herdecke (12/2016), we screened already available CBCT maxillary data from the years 2015–2016 (*n* = 547) of patients of various age groups and either sex, who had been treated at the dental clinic of the University of Witten/Herdecke. Patients with previous orthodontic treatment and with craniofacial or congenital abnormalities and syndromes such as cleft lip and palate, cysts or tumours in the maxilla were excluded from this study. Patients with insufficient image quality of the available CBCT were also excluded. Only 180 participants (95 females, 85 males, age range from 8 to 40 years) met the inclusion criteria and were divided into four different age groups (Table 1). The study was conducted according to the principles of the Declaration of Helsinki (1964) and its later amendments as well as in accordance with the current ethical guidelines and the ALARA principle.

**Table 1:** Patient distribution regarding age and sex

Group	Age in years	Female (n)	Male (n)	Total (n)
A	8–12	16	17	33
B	13–16	18	24	42
C	17–21	29	22	51
D	22–40	32	22	54
Total		95	85	180

### Radiological measurements

**Fig. 1:**
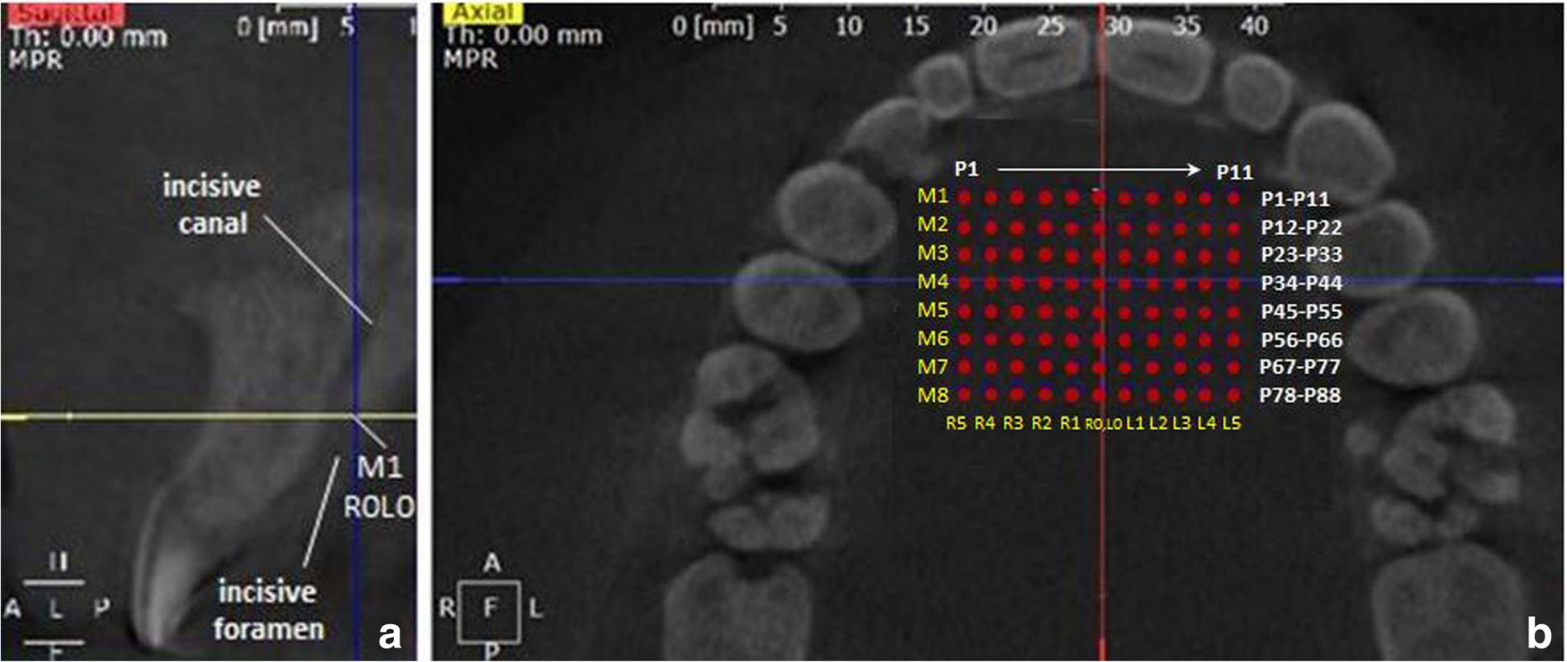
**a** Sagittal CBCT window depicting the alignment of the axial (yellow line) and coronal (blue line) planes for measurements. The crossing point in the median-sagittal plane denotes the location of the first palatal height measurement (M1, R0L0). **b** Axial CBCT window depicting the aligment of the sagittal plane (red line) for palatal bone height measurements, carried out according to a measurement grid (red dots), constructed starting from the posterior-cranial osseous border of the incisive foramen (M1, R0L0)

All CBCT images were generated in Digital Imaging and Communications in Medicine (DICOM) format with GALILEOS Comfort (Sirona Dental Systems GmbH, Germany) at an X-ray exposure of 85 kV and 5–7 mA (14 s, field of view: 150 × 150 mm, 200 singular images), yielding a voxel size of 0,027 mm^3^ and slice thickness of 300 μm. Patients were aligned according to the occlusal plane in the CBCT device. Patient data were anonymised by numerical designation of the subjects (A1-A33, B1-B42, C1-C51 and D1-D54) and only the date of birth and thus age and sex of the participants were disclosed to the investigator performing the measurements (R.V.) and the statistician (C.K.). CBCT analysis was performed in the bone window with the dds-pro® software (Digital Dental Service Ltd., England, version 1.4–2015) at a 23″ TFT-LCD monitor (resolution: 1920 × 1080 pixel at 60 Hz, contrast 1000:1, Philips V-line 236V3LSB6/00, Philips GmbH, Hamburg, Germany). Within the sagittal CBCT window (Fig. 1a), the axial (yellow line) and coronal (blue line) planes were aligned to the posterior-cranial osseous border of the incisive foramen (Foramen incisicum) and parallel/rectangular (axial/coronal plane) to the orthodontic occlusal plane (mesiopalatal cusp tips of the first molars, both cusp tips of the second and buccal cusp tips of the first premolars), whereas the sagittal plane (red line, Fig. 1b) was positioned according to the anterior and posterior nasal spine (median-sagittal plane). The standard point-to-point measurement tool was used to quantify palatal bone height at various points distanced 2 mm (± 0.01 mm) in sagittal (M1-M8) and transversal direction forming a measurement grid in axial view (Fig. 1b).

The first selected most anterior midline measurement point was the most cranial point of the posterior border of the incisive foramen (M1, R0L0, Fig. 1). From this point, additional 7 measurements were carried out in posterior sagittal direction at 2 mm (± 0.01 mm) intervals (M2-M8, R0L0). At each sagittal interval (M1-M8) 5 additional measurements were performed at 2 mm (± 0.01 mm) intervals in transversal direction to the right (R1–5) and left (L1–5) (Fig. 1b).

Altogether 88 grid points were selected and palatal bone height measured perpendicular to the axial plane either in sagittal or coronal view in 180 patients (CBCTs), who were divided into four age groups: group A (8–12 years), group B (13–16 years), group C (17–21 years) and group D (22–40 years). This resulted in a total of 15,840 measurements recorded in a Microsoft Excel data sheet (Microsoft Corporation, USA).

For 38 randomly selected patients of all age groups and either sex, all measurements were repeated by the same investigator (R.V.) after an appropriate time interval to assess intrarater reliability of CBCT measurements.

### Statistical analysis

Statistical analysis was performed using IBM® SPSS® Statistics 23 (IBM, Armonk, NY, USA). A descriptive exploratory data analysis was performed to verify the assumptions of parametric tests. Normal distribution within groups compared by significance tests was checked with Kolmogorov-Smirnov and Shapiro-Wilk tests as well as visually-optically by histograms. Variance homogeneity was assessed by Levene’s test and visually using zpred vs. zresid plots. As descriptive statistics arithmetic means (M) ± standard deviations (SD) as well as 95% bias corrected and accelerated confidence intervals (CI) of the mean (bootstrapping, 1000 samples) were calculated. For analytic statistics of the variable “palatal bone height”, parametric, mixed three-way analysis of variance (ANOVA) for the variables “sex”, “age group” and “measurement point at the palate (P1-P88)” was used, corrected according to Greenhouse-Geisser due to a violation of sphericity (Mauchly’s test), with pairwise post-hoc tests according to Hochberg’s GT2 due to differing group sizes. Significance level was set at *p* < 0.05. Intrarater reliability of CBCT measurements was determined by Lin’s concordance correlation coefficient CCC [25], which has considerable advantages over alternative methods and is robust with as few as 10 data pairs, with CCC > 0.99/0.95/0.9 denoting a perfect, substantial and moderate conformity of repeated measurements and thus intrarater reliability.

## Results

**Fig. 2:**
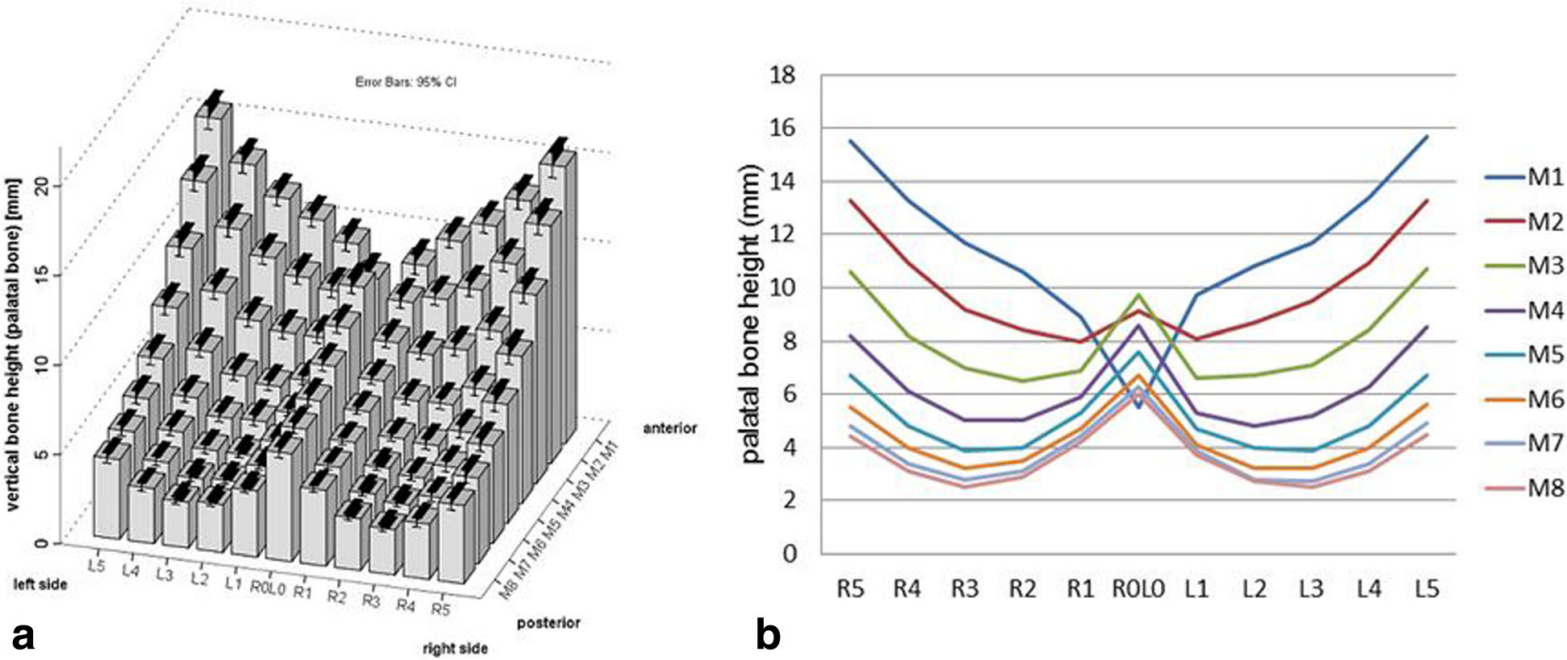
Palatal bone height (mm) at the 88 evaluated sites (P1-P88, a) and in anterior-posterior direction (M1-M8, b) for the total study population (*N* = 180). Mean (± 95% CI). Maximum mean bone height was found in the area of the raphe palatina mediana as well as in the anterior part of the palatal bone

The three-way ANOVA analysis for the variables “sex” and “age group” as well as “measurement point at the palate (P1-P88)” revealed significant differences in the target variable “palatal bone thickness (mm)” between the individual measuring points at the palate (P1-P88): F (9.88) = 485.657; *p* < 0.001 (Fig. 2, Additional file 1 Table S1). Maximum bone height was found in the area of ​​the midpalatal suture as well as in the anterior part of the palatal bone with the maximum at P28 (M3, R0L0, M ± SD 9.7 mm ± 2.8 mm). When examining the measuring points in sagittal direction, a decrease in vertical bone height was apparent from anterior (M3) to posterior (M8) direction.

**Fig. 3:**
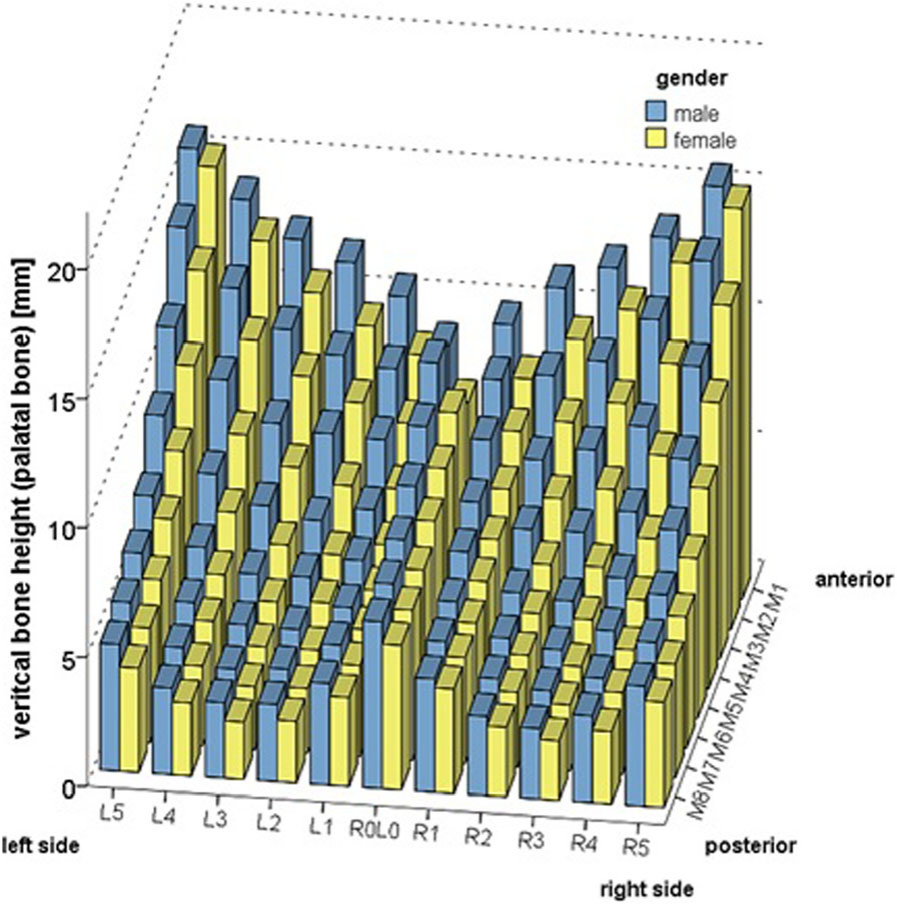
Mean palatal bone height (mm) at the 88 evaluated sites (P1-P88) for men and women. In general women show a lower bone height compared to men at all measuring points

Men and women showed significant differences in mean palatal bone height, which was generally lower in women at all measurement points: F(1) = 21.337; *p* < 0.001 (Fig. 3, Additional file 1 Tables S2 and S3).

**Fig. 4:**
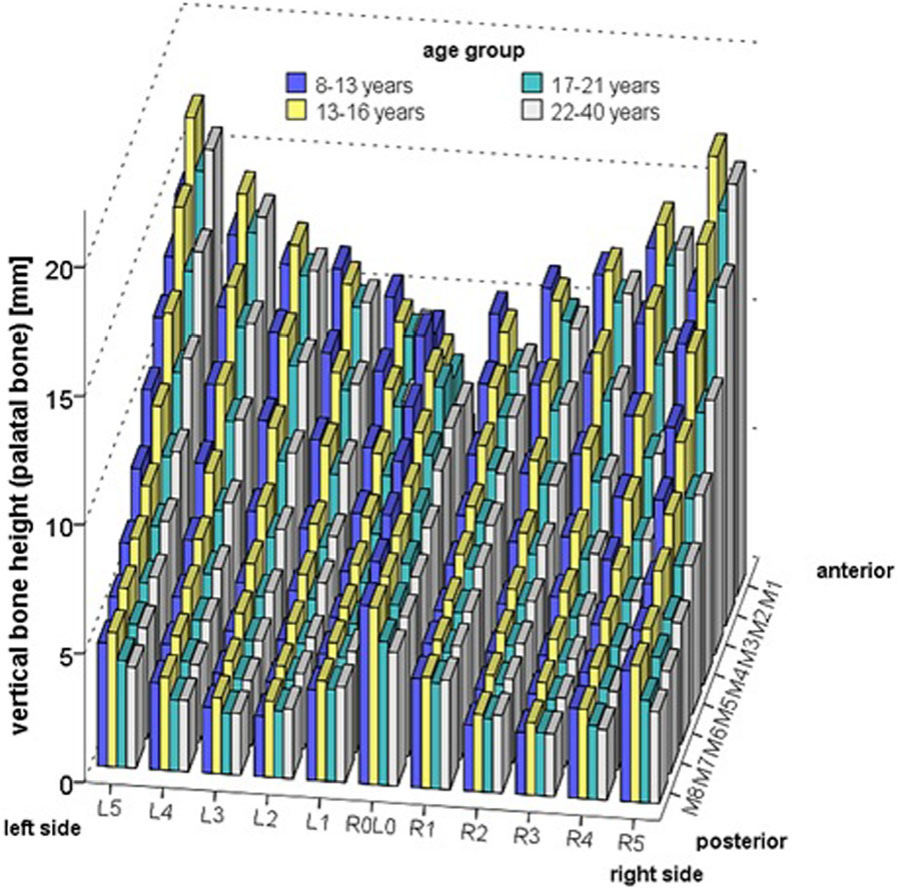
Mean palatal bone height (mm) at the 88 evaluated sites (P1-P88) for different age groups. In the area of the raphe palatina mediana as well as in the anterior and posterio-lateral area of the palate, there is generally a decrease in bone height with increasing age, whereas in the direction of the alveolar processes anterio-laterally between 13 and 16 years, there is a sharp increase, followed by a further decrease in bone height

Different age groups also differed significantly with respect to the mean height of the palatal bone: F(3) = 3.826; *p* = 0.011 (Fig. 4, Additional file 1 Tables S4–S7). In all age groups, maximum mean palatal bone height was found at P28 being 11.6 mm ± 3.1 mm (M ± SD) for group A (8–12 years), 10.3 mm ± 3.0 mm for group B (13–16 years), 9.7 mm ± 2.4 mm for group C (17–21 years) and 8.1 mm ± 2.3 mm for Group D (22–40 years). Pairwise comparisons for the variable “age group” revealed significant differences in mean palatal bone height between age groups 8–12 years and 17–21 years (*p* = 0.036, 95% CI of mean difference 0.4–2.0 mm), 13–16 years and 17–21 years (*p* = 0.004; 95% CI of mean difference 0.3–2.1 mm) as well as 13–16 years and 22–40 years (*p* = 0.033, 95% CI of mean difference: 0.5–1.8 mm).

Highly significant interactions of the variable “measurement point at the palate (P1-P88)” with the variable “sex” - F(9.88) = 2.976, *p* = 0.001 - as well as the variable “age group” - F(29.641) = 2.839, *p* < 0.001 - were found. This means that the significant differences in palatal bone height between different measurement points across the palate (P1-P88) differed for men and women as well as for individual age groups, i.e. sex and age have a significant impact on the extent of differences between measurement points.

**Fig. 5:**
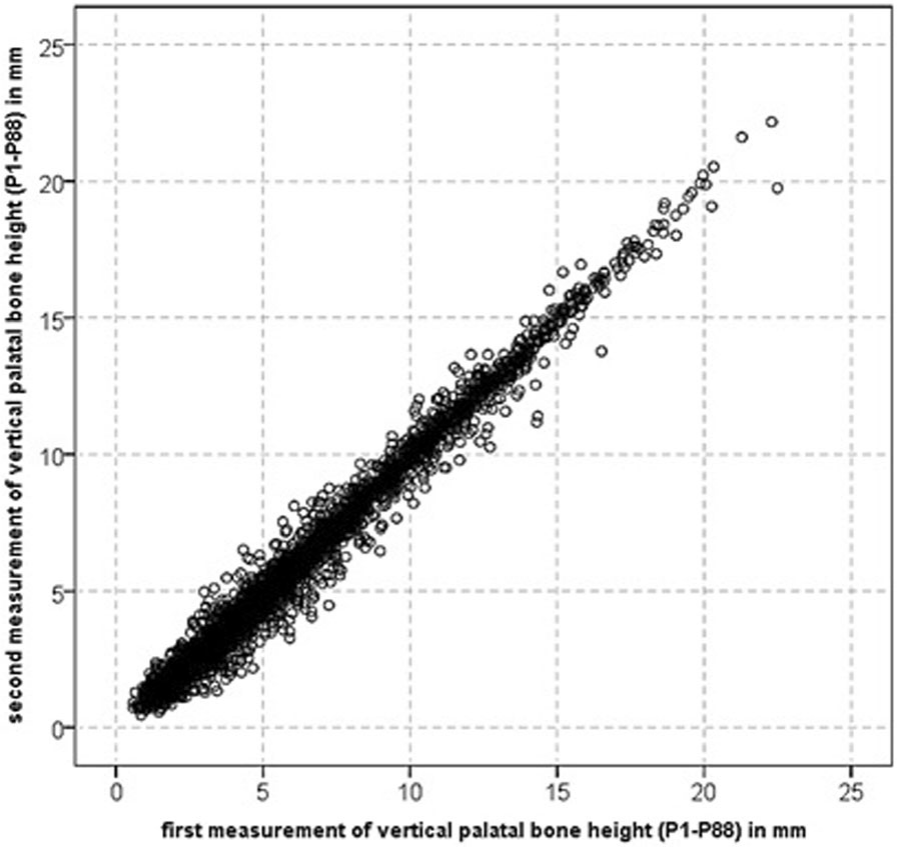
Palatal bone height (mm) at 88 evaluated sites for 38 patients (3344 paired single measurements). Intrarater agreement and thus intrarater reliability of measurements was substantial: CCC = 0.989 (95% CI 0.988–0.990)

Based on 3344 repeated single measurements (38 patients and 88 measurement points), a substantial intrarater agreement and thus intrarater reliability was corroborated: Lin’s CCC = 0.989 (95% CI 0.988–0.990) (Fig. 5).

## Discussion

The aim of the present study was to determine the optimal insertion site for orthodontic implants (OI), that is the area with maximum palatal bone height, in patients of different age and sex.

The findings of this study showed that the vertical palatal bone height decreases from anterior to posterior. In general, maximum mean height (9.7 mm ± 2.8 mm) was identified at the median palatal suture in particular at P28 (M3, R0L0), which is located 4 mm posterior to the incisive foramen. This is comparable to results reported by Gahleitner et al. based on CT scans of 32 patients aged 12–49 years, who found maximum mean palatal bone height (6.17 mm) 6 mm posterior to the incisive foramen [9]. Our results thus confirm previous findings, summarized by Winsauer et al. in a systematic review, that maximum mean palatal bone height ranging from 7.5–10.3 mm is located 3–4 mm posterior to the incisive foramen and within 5-8 mm lateral to the median palatal raphe [33]. In the present study palatal bone height ranged from 6.5 mm (± 2.3 mm) to 10.9 mm (± 3.4 mm) for the area 2 mm and 4 mm posterior to the incisive foramen and 4 mm, 6 mm, and 8 mm lateral to the median palatal raphe. Kang et al. found maximum mean palatal bone height only within 1 mm of ​​the midpalatal suture, which decreases from anterior to posterior and from medial to lateral direction, however, based on a limited sample of only 18 patients [18].

In the present study, we found significant sex variation in palatal bone height as well as significant differences among different age groups. These observations make sense regarding sexual dimorphism but also when considering the differing rate of physiological growth in puberty [5]. Bone turnover is strongly increased in puberty and sex steroids like growth hormone and insulin-like factors play a role in bone development [15, 29]. Boys have their puberal growth peak at about 14 years of age, whereas girls reach puberty 1.5–2 years earlier [22]. Thus, age group A was defined to cover the time before the puberal growth spurt in either boys or girls (8–12 years), whereas group B represented the maximum growth rate at 13–16 years and group C represent the age, when a decline of the growth occurs at 17–22 years. An additional group D was chosen as growth often continues beyond the age of 20 [13].

Our study showed regarding all measuring points that women presented lower vertical bone height for the individual age groups than men. The same sex specific pattern has been reported for other areas of the jaws like the alveolar bone, where women seem to have thinner bone compared to men [7].

Our findings indicate that palatal bone height is sex- and agespecific, which is of particular clinical relevance for the placement of orthodontic implants, when considering their acceptable length of insertion. In contrast to our study, some reports showed that women have an equal or greater bone supply in the posterior region of the palate than men [10–12, 18, 24, 31], whereas some studies confirm our results [15]. A possible reason for the discrepancy of study results could be differences in measuring procedures and protocol or the study population assessed with possible ethnic differences.

**Fig. 6:**
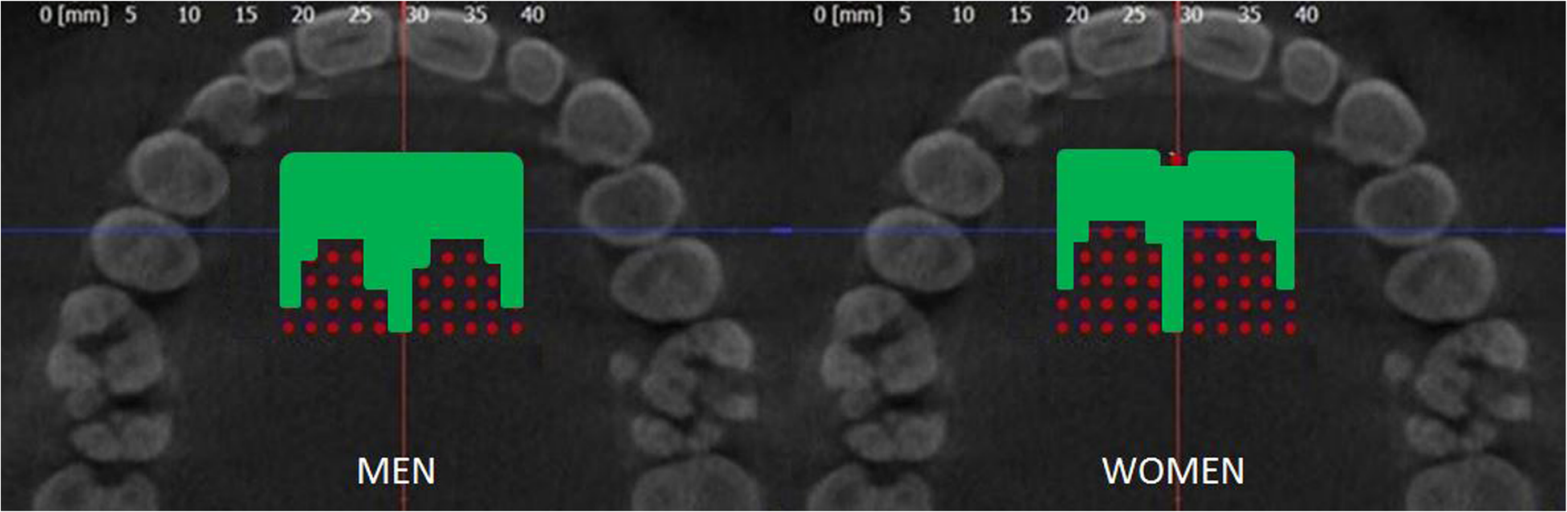
Optimal palatal insertions sites for orthodontic implants in men and women marked in green color

When clinically inserting an orthodontic implant (OI), a minimum of 4-5 mm of palatal bone height is required. According to the results of this study, suitable insertion sites of OI are points P1–46, P49-P51, P54-P56, P60, P61, P77, P67, P72, P77 and P83 in males, and points P1-P5, P7-P35, P38, P39, P43-P45, P50, P55, P56, P61, P66, P72, and P83 in females (Fig. 6). This means that the ideal insertion site is located ​​0-8 mm (men) or 0-6 mm (women) posterior to the incisive foramen and 10 mm lateral to the midpalatal suture. Despite these findings, careful diagnostics still have to be carried out for each individual patient before OI insertion, in particular, to avoid trauma to the incisive foramen bundle.

Based on the results of this study, a significant association between height of the palatal bone and age was determined. In the region of midpalatal suture as well as in the anterior and posterio lateral area of the palate, there is generally a well-defined decrease of palatal bone supply with advancing age. This could be explained by appositional growth of the maxilla up to the age of 15, which is accompanied by nasal resorption, which lasts even longer [28]. On the other hand, between 13 and 16 years of age a distinct increase of bone height followed by a later decrease was identified anterio-laterally, which corroborates previous findings [28]. Also the observed significant age differences in palatal bone height confirm previous findings [10, 21].

In this study, the incisive foramen was used as orientation point to establish a reproducible measurement grid of 280mm^3^, consisting of 88 grid points for reliable assessments of palatal bone height, as it provides a constant starting point for measurements in all patients [4, 10–12, 21, 31]. Compared to previous CBCT studies on palatal bone height, based on only 60–28 grid points and grid alignment according to highly variable dental structures (approximal contacts) [15, 16] as well as lower sample sizes [4, 9], this allowed higher precision and reliability of measurements as well as translatability to the clinical situation. Taking into consideration an OI diameter of 1.2-2 mm in case of non-osseointegrating mini-implants, the distance between points was set at 2 mm, enabling a highly sensitive measurement grid exploring all potential insertion areas [15]. Therefore, this study provides a more detailed view of palatal bone height distribution compared to other studies, which chose larger distances of 3-8 mm. Furthermore the total number of measurements performed (15,840) by far exceeds that of other studies ranging from 616 to 3240 points [4, 10, 21] with exception of Holm et al. measuring 25,860 points [15], explained by the higher number of patients assessed in their study (431).

## Conclusion

- Our findings demonstrate that vertical palatal bone height decreases from anterior to posterior direction.
- The midpalatal suture and the antero-lateral region of the palate provide sufficient bone height for insertion of orthodontic implants.
- A decrease in palatal bone height occurs at the palatal process of the maxilla with increasing age.
- From 13 to 16 years of age palatal alveolar bone height in lateral direction is increased.
- Palatal bone height in women is reduced compared to men.
- For optimal primary stability of orthodontic implants, the anterior palate as well as the midpalatal suture regions are favourable locations for implant insertion.

## Additional file

Additional file 1: **Table S1.** Palatal bone height (mm) at the 88 evaluated sites (P1-P88) for the total study population (*N* = 180). **Table S2.** Palatal bone height (mm) at the 88 evaluated sites (P1-P88) for men (*N* = 85). **Table S3.** Palatal bone height (mm) at the 88 evaluated sites (P1-P88) for women (*N* = 95). **Table S4.** Palatal bone height (mm) at the 88 evaluated sites (P1-P88) for age group 8-12 years (*N* = 33). **Table S5.** Palatal bone height (mm) at the 88 evaluated sites (P1-P88) for age group 13-16 years (*N* = 43). **Table S6.** Palatal bone height (mm) at the 88 evaluated sites (P1-P88) for age group 17-21 years (*N* = 51). **Table S7.** Palatal bone height (mm) at the 88 evaluated sites (P1-P88) for age group 22-40 years (*N* = 54). (DOCX 59 kb)
