# Correction to: Three-dimensional quantitative assessment of palatal bone height for insertion of orthodontic implants - a retrospective CBCT study

**DOI:** 10.1186/s13005-019-0198-4

**Published:** 2019-06-18

**Authors:** Sachin Chhatwani, Viola Rose-Zierau, Bassel Haddad, Mohammed Almuzian, Christian Kirschneck, Gholamreza Danesh

**Affiliations:** 1 Department of Orthodontics, University of Witten/Herdecke, Dental Clinic, Alfred-Herrhausen Str. 45, 58455 Witten, Germany; 2 Viola Rose-Zierau, Private Practice, Nelkenstr. 2, 44289 Dortmund, Germany; 3 Glasgow Dental Academy, Edinburgh, UK, Department of Orthodontics, University of Sydney, Sydney, NSW Australia; 4 Department of Orthodontics, University Medical Centre of Regensburg, Franz-Josef-Strauss-Allee 11, 93053 Regensburg, Germany

**Correction to: Head Face Med**

**https://doi.org/10.1186/s13005-019-0193-9**

Following publication of the original article [1], the authors reported that the information regarding ethics approval was accidentally entered under trial registration. A trial registration was not required for this study.
