# Supplementary material for: Three-dimensional quantitative assessment of palatal bone height for insertion of orthodontic implants - a retrospective CBCT study
Source: Head Face Med. 2019 Apr 1;15:9. doi: 10.1186/s13005-019-0193-9 (PMC6442434; doi:10.1186/s13005-019-0193-9)
Supplement: Supplementary file 1 — Table S1. Palatal bone height (mm) at the 88 evaluated sites (P1-P88) for the total study population (N = 180). Table S2. Palatal bone height (mm) at the 88 evaluated sites (P1-P88) for men (N = 85). Table S3. Palatal bone height (mm) at the 88 evaluated sites (P1-P88) for women (N = 95). Table S4. Palatal bone height (mm) at the 88 evaluated sites (P1-P88) for age group 8-12 years (N = 33). Table S5. Palatal bone height (mm) at the 88 evaluated sites (P1-P88) for age group 13-16 years (N = 43). Table S6. Palatal bone height (mm) at the 88 evaluated sites (P1-P88) for age group 17-21 years (N = 51). Table S7. Palatal bone height (mm) at the 88 evaluated sites (P1-P88) for age group 22-40 years (N = 54). (DOCX 59 kb) [file 13005_2019_193_MOESM1_ESM.docx]

**Supplementary Material**

Supplementary Table 1. Palatal bone height (mm) at the 88 evaluated sites (P1-P88) for the total study population (N = 180).

Supplementary Table 2. Palatal bone height (mm) at the 88 evaluated sites (P1-P88) for men (N = 85).

Supplementary Table 3. Palatal bone height (mm) at the 88 evaluated sites (P1-P88) for women (N = 95).

Supplementary Table 4. Palatal bone height (mm) at the 88 evaluated sites (P1-P88) for age group 8-12 years (N = 33).

Supplementary Table 5. Palatal bone height (mm) at the 88 evaluated sites (P1-P88) for age group 13-16 years (N = 43).

Supplementary Table 6. Palatal bone height (mm) at the 88 evaluated sites (P1-P88) for age group 17-21 years (N = 51).

Supplementary Table 7. Palatal bone height (mm) at the 88 evaluated sites (P1-P88) for age group 22-40 years (N = 54).

**Supplementary Material**

**Supplementary Table 1. Palatal bone height (mm) at the 88 evaluated sites (P1-P88) for the total study population (N = 180).** Indicated are the mean ± standard deviation and in parentheses the 95% bias-corrected and accelerated confidence interval of the mean (bootstrapping, 1,000 samples), which allows a prediction of the true population mean. Definition of measuring points P1-P88: see manuscript text.

| N=180 | **R5** | **R4** | **R3** | **R2** | **R1** | **R0L0** | **L1** | **L2** | **L3** | **L4** | **L5** |
| --- | --- | --- | --- | --- | --- | --- | --- | --- | --- | --- | --- |
| **M1** | P1  15.5 ± 4.6  (14.8 – 16.2) | P2  13.3 ± 3.6  (12.7 – 13.9) | P3  11.7 ± 3.1  (11.2 – 12.1) | P4  10.6 ± 2.9  (10.1 – 11.0) | P5  8.9 ± 3.8  (8.4 – 9.5) | P6  5.5 ± 3.0  (5.1 – 5.9) | P7  9.7 ± 3.3  (9.2 – 10.1) | P8  10.8 ± 3.0  (10.3 – 11.2) | P9  11.7 ± 3.2  (11.2 – 12.2) | P10  13.4 ± 3.7  (12.8 – 13.9) | P11  15.7 ± 4.4  (15.1 – 16.3) |
| **M2** | P12  13.3 ± 3.9  (12.7 – 13.9) | P13  10.9 ± 3.2  (10.4 – 11.4) | P14  9.2 ± 3.0  (8.8 – 9.6) | P15  8.4 ± 2.7  (8.1 – 8.8) | P16  8.0 ± 2.8  (7.7 – 8.4) | P17  9.1 ± 3.4  (8.6 – 9.7) | P18  8.1 ± 2.8  (7.7 – 8.6) | P19  8.7 ± 2.9  (8.2 – 9.0) | P20  9.5 ± 2.9  (9.1 – 9.9) | P21  10.9 ± 3.4  (10.3 – 11.4) | P22  13.3 ± 3.9  (12.7 – 13.8) |
| **M3** | P23  10.6 ± 3.8  (10.0 – 11.1) | P24  8.2 ± 3.1  (7.8 – 8.7) | P25  7.0 ± 2.6  (6.6 – 7.3) | P26  6.5 ± 2.3  (6.2 – 6.8) | P27  6.9 ± 2.4  (6.5 – 7.3) | P28  9.7 ± 2.9  (9.3 – 10.1) | P29  6.6 ± 2.3  (6.3 – 7.0) | P30  6.7 ± 2.6  (6.3 – 7.0) | P31  7.1 ± 2.8  (6.7 – 7.5) | P32  8.4 ± 2.9  (7.9 – 8.9) | P33  10.7 ± 3.5  (10.1 – 11.2) |
| **M4** | P34  8.2 ± 3.6  (7.7 – 8.8) | P35  6.1 ± 2.8  (5.7 – 6.5) | P36  5.0 ± 2.2  (4.7 – 5.3) | P37  5.0 ± 1.9  (4.7 – 5.3) | P38  5.9 ± 1.8  (5.6 – 6.1) | P39  8.6 ± 2.3  (8.3 – 8.9) | P40  5.3 ± 1.8  (5.1 – 5.6) | P41  4.8 ± 2.0  (4.5 – 5.1) | P42  5.2 ± 2.3  (4.8 – 5.5) | P43  6.3 ± 2.5  (5.9 – 6.6) | P44  8.5 ± 3.1  (8.0 – 8.9) |
| **M5** | P45  6.7 ± 3.3  (6.2 – 7.1) | P46  4.8 ± 2.6  (4.5 – 5.2) | P47  3.9 ± 2.0  (3.6 – 4.1) | P48  4.0 ± 1.5  (3.8 – 4.2) | P49  5.3 ± 1.5  (5.1 – 5.5) | P50  7.6 ± 2.3  (7.2 – 7.9) | P51  4.7 ± 1.5  (4.4 – 4.9) | P52  4.0 ± 2.1  (3.7 – 4.3) | P53  3.9 ± 1.9  (3.6 – 4.2) | P54  4.8 ± 2.2  (4.5 – 5.1) | P55  6.7 ± 2.9  (6.4 – 7.2) |
| **M6** | P56  5.5 ± 2.7  (5.1 – 5.9) | P57  4.0 ± 2.1  (3.7 – 4.3) | P58  3.2 ± 1.5  (3.0 – 3.4) | P59  3.5 ± 1.3  (3.3 – 3.7) | P60  4.7 ± 1.4  (4.5 – 4.9) | P61  6.7 ± 2.3  (6.4 – 7.1) | P62  4.1 ± 1.3  (3.9 – 4.3) | P63  3.2 ± 1.3  (3.0 – 3.4) | P64  3.2 ± 1.6  (3.0 – 3.4) | P65  4.0 ± 1.9  (3.7 – 4.3) | P66  5.6 ± 2.5  (5.3 – 6.0) |
| **M7** | P67  4.8 ± 2.4  (4.4 – 5.1) | P68  3.4 ± 1.8  (3.2 – 3.7) | P69  2.8 ± 1.2  (2.6 – 2.9) | P70  3.1 ± 1.2  (2.9 – 3.3) | P71  4.4 ± 1.3  (4.2 – 4.6) | P72  6.3 ± 2.2  (6.0 – 6.5) | P73  3.9 ± 1.3  (3.7 – 4.1) | P74  2.8 ± 1.2  (2.7 – 3.0) | P75  2.7 ± 1.3  (2.5 – 2.9) | P76  3.4 ± 1.6  (3.2 – 3.6) | P77  4.9 ± 2.1  (4.6 – 5.2) |
| **M8** | P78  4.4 ± 2.3  (4.1 – 4.7) | P79  3.1 ± 1.7  (2.8 – 3.3) | P80  2.5 ± 1.2  (2.3 – 2.7) | P81  2.9 ± 1.1  (2.7 – 3.0) | P82  4.2 ± 1.2  (4.0 – 4.4) | P83  6.0 ± 2.1  (5.7 – 6.3) | P84  3.7 ± 1.1  (3.5 – 3.8) | P85  2.7 ± 1.1  (2.5 – 2.8) | P86  2.5 ± 1.3  (2.4 – 2.7) | P87  3.1 ± 1.5  (2.9 – 3.3) | P88  4.5 ± 1.9  (4.2 – 4.7) |

**Supplementary Table 2. Palatal bone height (mm) at the 88 evaluated sites (P1-P88) for men (N = 85).** Indicated are the mean ± standard deviation and in parentheses the 95% bias-corrected and accelerated confidence interval of the mean (bootstrapping, 1,000 samples), which allows a prediction of the true population mean. Definition of measuring points P1-P88: see manuscript text.

| N=85 | **R5** | **R4** | **R3** | **R2** | **R1** | **R0L0** | **L1** | **L2** | **L3** | **L4** | **L5** |
| --- | --- | --- | --- | --- | --- | --- | --- | --- | --- | --- | --- |
| **M1** | P1  15.9 ± 4.7  (14.9 – 16.9) | P2  13.8 ± 3.7  (13.0 – 14.6) | P3  12.5 ± 2.8  (11.9 – 13.1) | P4  11.6 ± 2.5  (11.1 – 12.1) | P5  10.0 ± 3.6  (9.2 – 10.8) | P6  6.9 ± 3.6  (6.2 – 7.6) | P7  10.8 ± 3.0  (10.1 – 11.5) | P8  12.0 ± 2.5  (11.4 – 12.6) | P9  12.8 ± 3.0  (12.2 – 13.3) | P10  14.2 ± 3.4  (13.4 – 14.9) | P11  16.0 ± 4.8  (15.1 – 16.9) |
| **M2** | P12  14.2 ± 3.7  (13.4 – 14.9) | P13  11.8 ± 3.1  (11.2 – 12.5) | P14  10.0 ± 2.9  (9.4 – 10.6) | P15  9.4 ± 2.4  (8.8 – 9.8) | P16  9.1 ± 2.6  (8.5 – 9.7) | P17  10.2 ± 3.3  (9.5 – 11.0) | P18  9.2 ± 2.7  (8.7 – 9.8) | P19  9.6 ± 2.8  (9.0 – 10.2) | P20  10.4 ± 2.7  (9.9 – 11.0) | P21  11.9 ± 3.1  (11.3 – 12.5) | P22  14.1 ± 3.9  (13.2 – 15.0) |
| **M3** | P23  11.3 ± 3.7  (10.4 – 12.1) | P24  8.8 ± 3.1  (8.1 – 9.4) | P25  7.8 ± 2.6  (7.2 – 8.3) | P26  7.2 ± 2.3  (6.8 – 7.8) | P27  7.9 ± 2.3  (7.4 – 8.3) | P28  10.7 ± 2.8  (10.2 – 11.3) | P29  7.6 ± 2.3  (7.1 – 8.1) | P30  7.7 ± 2.5  (7.2 – 8.3) | P31  8.0 ± 2.8  (7.4 – 8.5) | P32  9.5 ± 2.8  (9.0 – 10.1) | P33  11.4 ± 3.6  (10.7 – 12.2) |
| **M4** | P34  8.8 ± 3.6  (8.1 – 9.6) | P35  6.6 ± 2.7  (6.1 – 7.3) | P36  5.7 ± 2.2  (5.3 – 6.1) | P37  5.7 ± 2.0  (5.3 – 6.1) | P38  6.6 ± 1.9  (6.2 – 7.0) | P39  9.4 ± 2.4  (8.9 – 9.9) | P40  6.0 ± 1.9  (5.7 – 6.5) | P41  5.5 ± 1.9  (5.1 – 5.9) | P42  5.9 ± 2.3  (5.4 – 6.5) | P43  7.0 ± 2.6  (6.5 – 7.5) | P44  9.2 ± 3.1  (8.5 – 9.8) |
| **M5** | P45  7.2 ± 3.2  (6.5 – 7.9) | P46  5.2 ± 2.3  (4.7 – 5.8) | P47  4.2 ± 1.9  (3.8 – 4.7) | P48  4.4 ± 1.6  (4.1 – 4.8) | P49  5.8 ± 1.5  (5.5 – 6.2) | P50  8.2 ± 2.3  (7.8 – 8.7) | P51  5.2 ± 1.5  (4.9 – 5.5) | P52  4.5 ± 1.8  (4.1 – 4.8) | P53  4.4 ± 2.0  (4.0 – 4.9) | P54  5.3 ± 2.3  (4.9 – 5.9) | P55  7.2 ± 2.9  (6.6 – 7.8) |
| **M6** | P56  5.9 ± 2.7  (5.3 – 6.4) | P57  4.3 ± 2.2  (3.9 – 4.8) | P58  3.5 ± 1.5  (3.2 – 3.8) | P59  3.8 ± 1.4  (3.5 – 4.1) | P60  5.1 ± 1.3  (4.8 – 5.3) | P61  7.3 ± 2.3  (6.8 – 7.8) | P62  4.6 ± 1.4  (4.3 – 4.9) | P63  3.6 ± 1.4  (3.3 – 3.9) | P64  3.6 ± 1.7  (3.3 – 4.0) | P65  4.4 ± 2.0  (3.9 – 4.8) | P66  6.1 ± 2.4  (5.5 – 6.7) |
| **M7** | P67  5.2 ± 2.5  (4.7 – 5.7) | P68  3.7 ± 1.9  (3.3 – 4.1) | P69  3.0 ± 1.3  (2.8 – 3.3) | P70  3.4 ± 1.2  (3.1 – 3.6) | P71  4.7 ± 1.2  (4.4 – 5.0) | P72  6.8 ± 2.3  (6.3 – 7.3) | P73  4.3 ± 1.5  (4.0 – 4.6) | P74  3.2 ± 1.3  (2.9 – 3.5) | P75  3.1 ± 1.4  (2.8 – 3.4) | P76  3.8 ± 1.7  (3.4 – 4.1) | P77  5.4 ± 2.2  (4.9 – 5.9) |
| **M8** | P78  4.7 ± 2.4  (4.2 – 5.2) | P79  3.4 ± 1.8  (3.0 – 3.7) | P80  2.7 ± 1.3  (2.5 – 3.0) | P81  3.1 ± 1.2  (2.8 – 3.3) | P82  4.4 ± 1.3  (4.1 – 4.6) | P83  6.5 ± 2.3  (6.0 – 6.9) | P84  3.9 ± 1.2  (3.7 – 4.2) | P85  3.0 ± 1.3  (2.7 – 3.3) | P86  2.9 ± 1.5  (2.6 – 3.3) | P87  3.3 ± 1.7  (3.0 – 3.7) | P88  4.9 ± 2.0  (4.5 – 5.4) |

**Supplementary Table 3. Palatal bone height (mm) at the 88 evaluated sites (P1-P88) for women (N = 95).** Indicated are the mean ± standard deviation and in parentheses the 95% bias-corrected and accelerated confidence interval of the mean (bootstrapping, 1,000 samples), which allows a prediction of the true population mean. Definition of measuring points P1-P88: see manuscript text.

| N=95 | **R5** | **R4** | **R3** | **R2** | **R1** | **R0L0** | **L1** | **L2** | **L3** | **L4** | **L5** |
| --- | --- | --- | --- | --- | --- | --- | --- | --- | --- | --- | --- |
| **M1** | P1  15.1 ± 4.5  (14.1 – 16.0) | P2  12.9 ± 3.5  (12.2 – 13.7) | P3  10.9 ± 3.2  (10.2 – 11.6) | P4  9.6 ± 3.0  (9.1 – 10.2) | P5  8.0 ± 3.7  (7.2 – 8.7) | P6  4.3 ± 1.6  (4.0 – 4.6) | P7  8.6 ± 3.1  (8.1 – 9.3) | P8  9.6 ± 2.9  (9.0 – 10.3) | P9  10.8 ± 3.0  (10.1 – 11.4) | P10  12.6 ± 3.7  (11.8 – 13.5) | P11  15.4 ± 4.0  (14.6 – 16.2) |
| **M2** | P12  12.5 ± 4.0  (11.7 – 13.3) | P13  10.1 ± 3.2  (9.5 – 10.8) | P14  8.5 ± 2.9  (7.9 – 9.1) | P15  7.6 ± 2.6  (7.0 – 8.2) | P16  7.1 ± 2.6  (6.6 – 7.6) | P17  8.1 ± 3.1  (7.5 – 8.7) | P18  7.1 ± 2.6  (6.7 – 7.7) | P19  7.8 ± 2.7  (7.3 – 8.3) | P20  8.7 ± 2.8  (8.1 – 9.3) | P21  9.9 ± 3.4  (9.2 – 10.7) | P22  12.5 ± 3.8  (11.8 – 13.3) |
| **M3** | P23  9.9 ± 3.7  (9.2 – 10.7) | P24  7.7 ± 3.1  (7.1 – 8.4) | P25  6.3 ± 2.5  (5.7 – 6.8) | P26  5.8 ± 2.2  (5.4 – 6.2) | P27  6.0 ± 2.1  (5.6 – 6.4) | P28  8.8 ± 2.7  (8.3 – 9.4) | P29  5.8 ± 1.9  (5.4 – 6.2) | P30  5.7 ± 2.2  (5.3 – 6.2) | P31  6.3 ± 2.5  (5.8 – 6.9) | P32  7.4 ± 2.6  (6.9 – 8.0) | P33  10.0 ± 3.3  (9.3 – 10.6) |
| **M4** | P34  7.7 ± 3.5  (7.1 – 8.6) | P35  5.6 ± 2.7  (5.1 – 6.2) | P36  4.4 ± 1.9  (4.0 – 4.8) | P37  4.4 ± 1.5  (4.1 – 4.7) | P38  5.2 ± 1.4  (4.9 – 5.5) | P39  7.8 ± 2.2  (7.4 – 8.3) | P40  4.7 ± 1.5  (4.5 – 5.0) | P41  4.2 ± 1.8  (3.9 – 4.5) | P42  4.5 ± 2.0  (4.1 – 4.9) | P43  5.6 ± 2.3  (5.1 – 6.1) | P44  7.8 ± 3.1  (7.3 – 8.4) |
| **M5** | P45  6.2 ± 3.3  (5.5 – 6.9) | P46  4.4 ± 2.7  (4.0 – 5.0) | P47  3.5 ± 2.0  (3.2 – 4.0) | P48  3.6 ± 1.3  (3.4 – 3.9) | P49  4.8 ± 1.3  (4.5 – 5.0) | P50  6.9 ± 2.2  (6.5 – 7.4) | P51  4.1 ± 1.3  (3.9 – 4.4) | P52  3.5 ± 2.2  (3.1 – 4.0) | P53  3.4 ± 1.6  (3.1 – 3.7) | P54  4.3 ± 2.0  (3.9 – 4.8) | P55  6.3 ± 2.9  (5.8 – 6.9) |
| **M6** | P56  5.1 ± 2.6  (4.6 – 5.6) | P57  3.7 ± 1.9  (3.3 – 4.0) | P58  2.9 ± 1.3  (2.6 – 3.1) | P59  3.2 ± 1.2  (3.0 – 3.4) | P60  4.3 ± 1.3  (4.0 – 4.5) | P61  6.2 ± 2.1  (5.8 – 6.7) | P62  3.8 ± 1.1  (3.6 – 4.0) | P63  2.8 ± 1.2  (2.6 – 3.1) | P64  2.8 ± 1.4  (2.6 – 3.1) | P65  3.7 ± 1.7  (3.4 – 4.0) | P66  5.2 ± 2.4  (4.7 – 5.7) |
| **M7** | P67  4.4 ± 2.4  (3.9 – 4.9) | P68  3.2 ± 1.6  (2.8 – 3.5) | P69  2.5 ± 1.2  (2.3 – 2.8) | P70  2.9 ± 1.2  (2.7 – 3.2) | P71  4.1 ± 1.3  (3.9 – 4.4) | P72  5.8 ± 1.9  (5.5 – 6.2) | P73  3.5 ± 1.0  (3.3 – 3.7) | P74  2.5 ± 1.0  (2.3 – 2.7) | P75  2.4 ± 1.1  (2.2 – 2.7) | P76  3.1 ± 1.4  (2.8 – 3.4) | P77  4.4 ± 1.9  (4.1 – 4.8) |
| **M8** | P78  4.1 ± 2.2  (3.6 – 4.5) | P79  2.8 ± 1.5  (2.5 – 3.1) | P80  2.3 ± 1.0  (2.1 – 2.5) | P81  2.7 ± 1.0  (2.5 – 2.9) | P82  4.0 ± 1.2  (3.8 – 4.3) | P83  5.6 ± 1.9  (5.3 – 6.0) | P84  3.4 ± 1.0  (3.3 – 3.6) | P85  2.4 ± 0.9  (2.2 – 2.5) | P86  2.2 ± 1.0  (2.0 – 2.4) | P87  2.8 ± 1.3  (2.6 – 3.1) | P88  4.0 ± 1.7  (3.7 – 4.4) |

**Supplementary Table 4. Palatal bone height (mm) at the 88 evaluated sites (P1-P88) for age group 8-12 years (N = 33).** Indicated are the mean ± standard deviation and in parentheses the 95% bias-corrected and accelerated confidence interval of the mean (bootstrapping, 1,000 samples), which allows a prediction of the true population mean. Definition of measuring points P1-P88: see manuscript text.

| N=33 | **R5** | **R4** | **R3** | **R2** | **R1** | **R0L0** | **L1** | **L2** | **L3** | **L4** | **L5** |
| --- | --- | --- | --- | --- | --- | --- | --- | --- | --- | --- | --- |
| **M1** | P1  13.4 ± 6.9  (11.1 – 15.7) | P2  13.3 ± 4.4  (11.5 – 14.8) | P3  12.1 ± 3.4  (10.7 – 13.3) | P4  11.5 ± 2.7  (10.4 – 12.4) | P5  10.3 ± 3.8  (8.9 – 11.7) | P6  6.3 ± 4.1  (5.0 – 7.8) | P7  10.7 ± 3.3  (9.5 – 11.8) | P8  11.7 ± 2.8  (10.7 – 12.6) | P9  11.7 ± 3.8  (10.3 – 12.9) | P10  12.7 ± 4.7  (10.9 – 14.3) | P11  14.2 ± 6.1  (11.7 – 16.2) |
| **M2** | P12  12.9 ± 4.8  (11.2 – 14.4) | P13  11.5 ± 3.1  (10.2 – 12.6) | P14  9.5 ± 3.0  (8.3 – 10.5) | P15  8.9 ± 2.4  (8.1 – 9.7) | P16  8.8 ± 3.0  (7.8 – 9.8) | P17  10.6 ± 4.6  (9.0 – 12.3) | P18  9.0 ± 3.0  (7.9 – 10.1) | P19  9.6 ± 3.0  (8.5 – 10.6) | P20  10.2 ± 3.0  (9.3 – 10.8) | P21  11.0 ± 3.4  (9.9 – 12.2) | P22  12.9 ± 4.4  (11.4 – 14.3) |
| **M3** | P23  12.0 ± 3.6  (10.9 – 13.0) | P24  9.1 ± 3.0  (8.1 – 10.1) | P25  7.5 ± 2.5  (6.7 – 8.3) | P26  6.6 ± 1.9  (5.9 – 7.2) | P27  7.1 ± 2.2  (6.4 – 7.8) | P28  11.6 ± 3.1  (10.5 – 12.7) | P29  7.2 ± 2.5  (6.4 – 8.0) | P30  7.3 ± 2.9  (6.4 – 8.4) | P31  7.9 ± 2.6  (7.1 – 9.0) | P32  9.2 ± 2.5  (8.4 – 10.1) | P33  11.7 ± 3.7  (10.5 – 12.8) |
| **M4** | P34  9.9 ± 4.0  (8.6 – 11.5) | P35  7.1 ± 3.0  (6.2 – 7.9) | P36  5.4 ± 2.2  (4.8 – 6.1) | P37  5.0 ± 1.9  (4.5 – 5.7) | P38  5.9 ± 1.5  (5.4 – 6.5) | P39  10.0 ± 2.6  (9.1 – 10.9) | P40  5.8 ± 1.8  (5.2 – 6.3) | P41  5.0 ± 2.2  (4.6 – 5.9) | P42  5.6 ± 2.6  (4.7 – 6.4) | P43  7.3 ± 2.7  (6.5 – 8.2) | P44  10.0 ± 3.3  (8.9 – 11.3) |
| **M5** | P45  8.2 ± 3.4  (7.2 – 9.4) | P46  5.8 ± 3.4  (4.8 – 7.0) | P47  4.2 ± 2.6  (3.5 – 5.2) | P48  3.8 ± 1.3  (3.4 – 4.3) | P49  5.2 ± 1.2  (4.8 – 5.6) | P50  9.1 ± 2.5  (8.2 – 10.0) | P51  4.7 ± 1.4  (4.2 – 5.1) | P52  3.8 ± 1.7  (3.2 – 4.4) | P53  4.0 ± 1.8  (3.4 – 4.6) | P54  5.5 ± 2.4  (4.8 – 6.2) | P55  8.1 ± 3.6  (6.9 – 9.4) |
| **M6** | P56  6.1 ± 2.8  (5.3 – 7.1) | P57  4.4 ± 2.0  (3.8 – 5.2) | P58  3.1 ± 1.4  (2.7 – 3.5) | P59  3.1 ± 1.3  (2.7 – 3.5) | P60  4.6 ± 1.5  (4.1 – 5.1) | P61  8.1 ± 2.9  (7.2 – 9.0) | P62  4.0 ± 1.1  (3.7 – 4.4) | P63  3.0 ± 1.3  (2.5 – 3.5) | P64  3.2 ± 1.6  (2.7 – 3.7) | P65  4.4 ± 2.0  (3.7 – 5.1) | P66  6.4 ± 3.0  (5.4 – 7.4) |
| **M7** | P67  5.5 ± 2.7  (4.6 – 6.5) | P68  3.7 ± 1.8  (3.1 – 4.4) | P69  2.7 ± 1.2  (2.4 – 3.1) | P70  2.8 ± 1.3  (2.4 – 3.2) | P71  4.4 ± 1.6  (3.9 – 5.0) | P72  7.5 ± 2.7  (6.6 – 8.5) | P73  3.7 ± 1.0  (3.4 – 4.1) | P74  2.5 ± 1.1  (2.2 – 2.9) | P75  2.7 ± 1.4  (2.2 – 3.2) | P76  3.7 ± 1.9  (3.2 – 4.4) | P77  5.4 ± 2.6  (4.6 – 6.3) |
| **M8** | P78  5.1 ± 2.2  (4.3 – 5.8) | P79  3.5 ± 1.9  (2.9 – 4.0) | P80  2.4 ± 1.2  (2.0 – 2.8) | P81  2.5 ± 1.1  (2.2 – 2.9) | P82  4.2 ± 1.2  (3.9 – 4.6) | P83  6.9 ± 2.6  (6.1 – 7.8) | P84  3.5 ± 0.8  (3.2 – 3.8) | P85  2.3 ± 0.9  (2.1 – 2.6) | P86  2.6 ± 1.3  (2.2 – 3.0) | P87  3.4 ± 1.8  (2.8 – 3.9) | P88  4.8 ± 2.2  (4.1 – 5.6) |

**Supplementary Table 5. Palatal bone height (mm) at the 88 evaluated sites (P1-P88) for age group 13-16 years (N = 43).** Indicated are the mean ± standard deviation and in parentheses the 95% bias-corrected and accelerated confidence interval of the mean (bootstrapping, 1,000 samples), which allows a prediction of the true population mean. Definition of measuring points P1-P88: see manuscript text.

| N=42 | **R5** | **R4** | **R3** | **R2** | **R1** | **R0L0** | **L1** | **L2** | **L3** | **L4** | **L5** |
| --- | --- | --- | --- | --- | --- | --- | --- | --- | --- | --- | --- |
| **M1** | P1  17.0 ± 3.3  (16.0 – 17.9) | P2  14.2 ± 2.9  (13.5 – 15.0) | P3  12.3 ± 3.1  (11.3 – 13.3) | P4  11.5 ± 2.7  (10.4 – 12.4) | P5  9.6 ± 4.0  (8.5 – 10.7) | P6  5.8 ± 2.9  (5.0 – 6.6) | P7  9.8 ± 3.2  (8.7 – 10.8) | P8  11.1 ± 2.8  (10.3 – 11.9) | P9  12.5 ± 2.7  (11.7 – 13.3) | P10  14.3 ± 2.9  (13.4 – 15.2) | P11  17.2 ± 3.3  (16.2 – 18.0) |
| **M2** | P12  14.8 ± 3.5  (13.7 – 15.9) | P13  12.1 ± 3.3  (11.1 – 13.2) | P14  10.3 ± 2.9  (9.4 – 11.2) | P15  9.0 ± 2.8  (8.1 – 9.8) | P16  8.7 ± 2.9  (7.8 – 9.5) | P17  9.7 ± 3.6  (8.7 – 10.8) | P18  8.3 ± 2.7  (7.6 – 9.1) | P19  8.8 ± 3.2  (7.8 – 9.8) | P20  10.1 ± 2.9  (9.3 – 10.8) | P21  11.9 ± 3.1  (11.0 – 12.7) | P22  14.8 ± 3.4  (13.8 – 15.9) |
| **M3** | P23  11.7 ± 3.5  (10.6 – 12.7) | P24  9.1 ± 3.3  (8.1 – 10.2) | P25  7.7 ± 2.9  (6.9 – 8.6) | P26  7.1 ± 2.7  (6.3 – 8.0) | P27  7.5 ± 2.6  (6.8 – 8.3) | P28  10.3 ± 3.0  (9.5 – 11.1) | P29  7.0 ± 2.6  (6.1 – 7.8) | P30  7.1 ± 2.8  (6.4 – 8.0) | P31  7.7 ± 2.9  (6.8 – 8.6) | P32  9.2 ± 3.0  (8.4 – 10.1) | P33  11.9 ± 3.2  (8.7 – 10.5) |
| **M4** | P34  9.4 ± 3.5  (8.4 – 10.5) | P35  7.0 ± 2.8  (6.2 – 7.9) | P36  5.6 ± 2.4  (4.9 – 6.4) | P37  5.4 ± 2.2  (4.8 – 6.1) | P38  6.3 ± 2.1  (5.7 – 7.0) | P39  9.1 ± 2.7  (8.3 – 9.8) | P40  5.6 ± 2.1  (5.0 – 6.3) | P41  5.2 ± 2.0  (4.6 – 5.9) | P42  5.8 ± 2.3  (5.1 – 6.4) | P43  7.0 ± 2.4  (6.3 – 7.6) | P44  9.4 ± 2.8  (8.6 – 10.3) |
| **M5** | P45  7.7 ± 3.4  (6.8 – 8.6) | P46  5.4 ± 2.4  (4.7 – 6.2) | P47  4.5 ± 1.9  (3.9 – 5.0) | P48  4.4 ± 1.7  (3.9 – 4.9) | P49  5.6 ± 1.6  (5.1 – 6.1) | P50  8.1 ± 2.5  (7.4 – 8.9) | P51  5.0 ± 1.7  (4.4 – 5.5) | P52  4.4 ± 1.9  (3.9 – 5.0) | P53  4.7 ± 2.1  (4.2 – 5.3) | P54  5.5 ± 2.2  (4.9 – 1.5) | P55  7.5 ± 2.6  (6.7 – 8.2) |
| **M6** | P56  6.7 ± 2.8  (5.9 – 7.5) | P57  4.7 ± 2.4  (4.0 – 5.4) | P58  3.7 ± 1.7  (3.3 – 4.3) | P59  3.9 ± 1.4  (3.4 – 4.3) | P60  5.0 ± 1.4  (4.6 – 5.4) | P61  7.3 ± 2.5  (6.6 – 8.1) | P62  4.5 ± 1.4  (4.1 – 4.8) | P63  3.7 ± 1.3  (3.3 – 4.1) | P64  3.9 ± 1.6  (3.5 – 4.4) | P65  4.8 ± 1.8  (4.3 – 5.3) | P66  6.6 ± 2.2  (5.9 – 7.2) |
| **M7** | P67  5.6 ± 2.4  (4.9 – 6.3) | P68  3.9 ± 1.8  (3.4 – 4.5) | P69  3.2 ± 1.3  (2.8 – 3.6) | P70  3.4 ± 1.1  (3.1 – 3.7) | P71  4.6 ± 1.1  (4.2 – 4.9) | P72  6.9 ± 2.5  (6.2 – 7.7) | P73  4.2 ± 1.6  (3.8 – 4.7) | P74  3.2 ± 1.3  (2.9 – 3.5) | P75  3.2 ± 1.4  (2.9 – 3.6) | P76  4.1 ± 1.5  (3.7 – 4.5) | P77  5.8 ± 1.9  (5.3 – 6.3) |
| **M8** | P78  5.3 ± 2.5  (4.6 – 6.2) | P79  3.5 ± 1.8  (3.0 – 4.0) | P80  2.8 ± 1.3  (2.5 – 3.2) | P81  3.0 ± 1.2  (2.5 – 3.2) | P82  4.3 ± 1.2  (4.0 – 4.6) | P83  6.9 ± 2.6  (6.1 – 7.7) | P84  3.9 ± 1.4  (3.4 – 4.4) | P85  3.0 ± 1.4  (2.6 – 3.4) | P86  2.9 ± 1.4  (2.6 – 3.3) | P87  3.6 ± 1.6  (3.2 – 4.0) | P88  5.2 ± 1.9  (4.7 – 5.8) |

**Supplementary Table 6. Palatal bone height (mm) at the 88 evaluated sites (P1-P88) for age group 17-21 years (N = 51).** Indicated are the mean ± standard deviation and in parentheses the 95% bias-corrected and accelerated confidence interval of the mean (bootstrapping, 1,000 samples), which allows a prediction of the true population mean. Definition of measuring points P1-P88: see manuscript text.

| N=51 | **R5** | **R4** | **R3** | **R2** | **R1** | **R0L0** | **L1** | **L2** | **L3** | **L4** | **L5** |
| --- | --- | --- | --- | --- | --- | --- | --- | --- | --- | --- | --- |
| **M1** | P1  14.9 ± 4.4  (13.4 – 16.4) | P2  12.7 ± 3.7  (11.5 – 13.7) | P3  11.1 ± 3.1  (10.3 – 11.9) | P4  10.2 ± 2.7  (9.5 – 11.0) | P5  8.1 ± 3.1  (7.3 – 9.0) | P6  5.5 ± 2.7  (4.8 – 6.2) | P7  9.2 ± 2.7  (8.4 – 10.1) | P8  10.2 ± 2.8  (9.4 – 11.0) | P9  11.3 ± 3.2  (10.4 – 12.2) | P10  12.8 ± 3.7  (11.8 – 13.9) | P11  15.1 ± 4.6  (13.8 – 16.4) |
| **M2** | P12  12.5 ± 3.6  (11.5 – 13.5) | P13  10.0 ± 3.0  (9.1 – 10.8) | P14  8.4 ± 3.0  (7.7 – 9.3) | P15  7.9 ± 2.8  (7.1 – 8.8) | P16  7.5 ± 2.4  (6.9 – 8.2) | P17  9.0 ± 2.5  (8.2 – 9.7) | P18  7.6 ± 2.3  (7.0 – 8.3) | P19  8.2 ± 2.5  (7.4 – 8.9) | P20  9.0 ± 2.8  (8.2 – 9.7) | P21  10.3 ± 3.3  (9.3 – 11.3) | P22  12.4 ± 4.0  (11.1 – 13.7) |
| **M3** | P23  9.4 ± 3.8  (8.4 – 10.4) | P24  7.5 ± 3.0  (6.8 – 8.3) | P25  6.4 ± 2.5  (5.7 – 7.2) | P26  6.2 ± 2.4  (5.6 – 6.8) | P27  6.6 ± 2.3  (6.0 – 7.3) | P28  9.7 ± 2.4  (9.2 – 10.2) | P29  6.1 ± 1.8  (5.6 – 6.7) | P30  6.0 ± 2.2  (5.4 – 6.6) | P31  6.4 ± 2.6  (5.7 – 7.3) | P32  7.8 ± 2.9  (7.0 – 8.6) | P33  9.6 ± 3.3  (8.7 – 10.5) |
| **M4** | P34  7.2 ± 3.3  (6.3 – 8.1) | P35  5.4 ± 2.4  (4.7 – 6.2) | P36  4.6 ± 1.9  (4.1 – 5.0) | P37  4.6 ± 1.7  (4.2 – 5.1) | P38  5.7 ± 1.8  (5.2 – 6.2) | P39  8.2 ± 1.8  (7.8 – 8.7) | P40  5.0 ± 1.4  (4.7 – 5.3) | P41  4.4 ± 1.8  (3.9 – 4.9) | P42  4.6 ± 2.0  (3.9 – 5.2) | P43  5.5 ± 2.3  (4.8 – 6.2) | P44  7.5 ± 2.9  (6.5 – 8.4) |
| **M5** | P45  5.7 ± 3.1  (5.1 – 6.5) | P46  4.3 ± 2.2  (3.7 – 4.8) | P47  3.5 ± 1.7  (3.1 – 3.9) | P48  3.8 ± 1.5  (3.5 – 4.2) | P49  5.1 ± 1.5  (4.7 – 5.6) | P50  7.1 ± 1.8  (6.7 – 7.6) | P51  4.3 ± 1.3  (3.9 – 4.6) | P52  3.5 ± 1.5  (3.1 – 3.9) | P53  3.4 ± 1.7  (3.0 – 3.9) | P54  4.2 ± 2.0  (3.7 – 4.7) | P55  5.9 ± 2.5  (5.2 – 6.6) |
| **M6** | P56  4.8 ± 2.5  (4.2 – 5.5) | P57  3.6 ± 1.8  (3.2 – 4.1) | P58  2.9 ± 1.3  (2.6 – 3.3) | P59  3.4 ± 1.3  (3.0 – 3.7) | P60  4.5 ± 1.2  (4.2 – 4.9) | P61  6.3 ± 1.4  (5.9 – 6.8) | P62  3.8 ± 1.2  (3.5 – 4.2) | P63  2.9 ± 1.2  (2.6 – 3.3) | P64  2.9 ± 1.5  (2.5 – 3.3) | P65  3.6 ± 1.7  (3.1 – 4.0) | P66  4.9 ± 2.1  (4.3 – 5.5) |
| **M7** | P67  4.5 ± 2.4  (3.9 – 5.2) | P68  3.1 ± 1.6  (2.7 – 3.6) | P69  2.6 ± 1.3  (2.3 – 2.9) | P70  3.0 ± 1.2  (2.7 – 3.3) | P71  4.1 ± 1.2  (3.8 – 4.4) | P72  5.8 ± 1.6  (5.4 – 6.3) | P73  3.7 ± 1.2  (3.3 – 4.0) | P74  2.7 ± 1.2  (2.3 – 3.0) | P75  2.6 ± 1.2  (2.3 – 2.9) | P76  3.1 ± 1.5  (2.7 – 3.5) | P77  4.4 ± 1.9  (3.9 – 4.9) |
| **M8** | P78  4.0 ± 2.4  (3.4 – 4.6) | P79  2.8 ± 1.6  (2.5 – 3.2) | P80  2.4 ± 1.1  (2.1 – 2.8) | P81  2.8 ± 1.1  (2.5 – 3.2) | P82  4.1 ± 1.1  (3.8 – 4.4) | P83  5.6 ± 1.5  (5.2 – 6.0) | P84  3.6 ± 1.1  (3.3 – 3.9) | P85  2.6 ± 1.1  (2.3 – 2.9) | P86  2.4 ± 1.1  (2.0 – 2.8) | P87  2.7 ± 1.3  (2.4 – 3.1) | P88  4.2 ± 1.8  (3.7 – 4.6) |

**Supplementary Table 7. Palatal bone height (mm) at the 88 evaluated sites (P1-P88) for age group 22-40 years (N = 54).** Indicated are the mean ± standard deviation and in parentheses the 95% bias-corrected and accelerated confidence interval of the mean (bootstrapping, 1,000 samples), which allows a prediction of the true population mean. Definition of measuring points P1-P88: see manuscript text.

| N=54 | **R5** | **R4** | **R3** | **R2** | **R1** | **R0L0** | **L1** | **L2** | **L3** | **L4** | **L5** |
| --- | --- | --- | --- | --- | --- | --- | --- | --- | --- | --- | --- |
| **M1** | P1  16.0 ± 3.6  (15.1 – 16.9) | P2  13.3 ± 3.4  (12.4 – 14.3) | P3  11.5 ± 2.9  (10.7 – 12.2) | P4  9.9 ± 3.0  (9.1 – 10.9) | P5  8.4 ± 4.0  (7.4 – 9.4) | P6  4.8 ± 2.5  (4.2 – 5.4) | P7  9.4 ± 3.7  (8.3 – 10.3) | P8  10.2 ± 2.9  (9.4 – 11.0) | P9  11.5 ± 3.0  (10.8 – 12.4) | P10  13.5 ± 3.4  (12.6 – 14.4) | P11  16.0 ± 3.5  (15.1 – 17.0) |
| **M2** | P12  13.1 ± 3.7  (12.2 – 14.1) | P13  10.5 ± 3.2  (9.6 – 11.3) | P14  8.9 ± 2.8  (8.2 – 9.7) | P15  8.2 ± 2.6  (7.5 – 9.0) | P16  7.5 ± 2.7  (6.8 – 8.3) | P17  7.9 ± 2.6  (7.2 – 8.6) | P18  7.6 ± 2.3  (7.0 – 8.3) | P19  8.2 ± 2.5  (7.4 – 8.9) | P20  9.0 ± 2.8  (8.2 – 9.7) | P21  10.5 ± 3.5  (9.5 – 11.5) | P22  13.1 ± 3.6  (12.1 – 14.2) |
| **M3** | P23  9.9 ± 3.6  (9.0 – 10.8) | P24  7.7 ± 3.0  (6.9 – 8.5) | P25  6.6 ± 2.5  (6.0 – 7.3) | P26  6.2 ± 2.2  (5.6 – 6.8) | P27  6.5 ± 2.3  (5.9 – 7.2) | 5P28  8.1 ± 2.3  (7.5 – 8.8) | P29  6.5 ± 2.1  (5.9 – 7.1) | P30  6.5 ± 2.4  (5.9 – 7.1) | P31  6.8 ± 2.7  (6.1 – 7.4) | P32  7.9 ± 3.0  (7.2 – 8.6) | P33  10.2 ± 3.6  (9.2 – 11.1) |
| **M4** | P34  7.4 ± 3.0  (6.5 – 8.2) | P35  5.5 ± 2.6  (4.8 – 6.1) | P36  4.8 ± 2.1  (4.2 – 5.4) | P37  5.0 ± 1.8  (4.5 – 5.5) | P38  5.6 ± 1.6  (5.2 – 6.1) | P39  7.6 ± 2.2  (7.1 – 8.2) | P40  5.2 ± 1.9  (4.7 – 5.8) | P41  4.8 ± 1.9  (4.3 – 5.2) | P42  4.9 ± 2.1  (4.3 – 5.6) | P43  5.8 ± 2.4  (5.3 – 6.5) | P44  7.7 ± 3.0  (6.9 – 8.5) |
| **M5** | P45  5.8 ± 2.7  (5.1 – 6.6) | P46  4.3 ± 2.3  (3.7 – 4.9) | P47  3.6 ± 1.7  (3.1 – 4.0) | P48  4.0 ± 1.4  (3.7 – 4.3) | P49  5.2 ± 1.5  (4.8 – 5.6) | P50  6.6 ± 2.0  (6.1 – 7.0) | P51  4.8 ± 1.5  (4.4 – 5.2) | P52  4.2 ± 2.8  (3.5 – 5.0) | P53  3.7 ± 1.8  (3.2 – 4.1) | P54  4.4 ± 2.1  (3.9 – 5.0) | P55  6.1 ± 2.6  (5.5 – 6.8) |
| **M6** | P56  4.7 ± 2.3  (4.1 – 5.3) | P57  3.5 ± 2.0  (3.0 – 4.0) | P58  3.0 ± 1.4  (2.7 – 3.4) | P59  3.5 ± 1.3  (3.1 – 3.8) | P60  4.6 ± 1.4  (4.2 – 5.0) | P61  5.8 ± 1.8  (5.4 – 6.2) | P62  4.3 ± 1.4  (3.9 – 4.7) | P63  3.2 ± 1.4  (2.8 – 3.5) | P64  2.9 ± 1.6  (2.5 – 3.4) | P65  3.6 ± 1.8  (3.1 – 4.1) | P66  5.1 ± 2.3  (4.6 – 5.7) |
| **M7** | P67  4.0 ± 2.0  (3.4 – 4.5) | P68  3.0 ± 1.7  (2.6 – 3.5) | P69  2.7 ± 1.2  (2.3 – 3.0) | P70  3.3 ± 1.3  (2.9 – 3.6) | P71  4.4 ± 1.3  (4.1 – 4.8) | P72  5.4 ± 1.4  (5.1 – 5.8) | P73  3.9 ± 1.3  (3.6 – 4.3) | P74  2.9 ± 1.2  (2.6 – 3.2) | P75  2.5 ± 1.3  (2.2 – 2.8) | P76  3.0 ± 1.5  (2.6 – 3.4) | P77  4.3 ± 1.9  (3.8 – 4.8) |
| **M8** | P78  3.5 ± 1.7  (3.1 – 4.0) | P79  2.7 ± 1.5  (2.3 – 3.2) | P80  2.4 ± 1.2  (2.1 – 2.7) | P81  3.0 ± 1.1  (2.7 – 3.3) | P82  4.2 ± 1.4  (3.9 – 4.6) | P83  5.2 ± 1.3  (4.9 – 5.5) | P84  3.7 ± 1.0  (3.4 – 3.9) | P85  2.7 ± 1.1  (2.4 – 3.0) | P86  2.4 ± 1.2  (2.1 – 2.8) | P87  2.8 ± 1.3  (2.4 – 3.0) | P88  3.9 ± 1.6  (3.5 – 4.3) |
